# Manganese-Based Oxide Cathode Materials for Aqueous Magnesium-Ion Batteries

**DOI:** 10.3390/molecules31122165

**Published:** 2026-06-19

**Authors:** Fangyu Xiong, Yixin Li, Xiaolin Zhang, Bo Liu, Yaohong Yang, Guangsheng Huang, Paul K. Chu

**Affiliations:** 1 National Engineering Research Center for Magnesium Alloys, College of Materials Science and Engineering, Chongqing University, Chongqing 400044, China; 2 Department of Physics, City University of Hong Kong, Tat Chee Avenue, Kowloon, Hong Kong, China

**Keywords:** aqueous battery, magnesium-ion battery, manganese-based oxide, cathode material

## Abstract

Aqueous magnesium-ion batteries (AMIBs) are promising for next-generation energy storage technologies due to their high safety, low cost, high theoretical energy density, and environmental friendliness. In particular, manganese-based oxides have attracted much attention due to the abundant resources, high theoretical capacity, and environmental friendliness. This paper provides a comprehensive overview of manganese-based oxide cathode materials for AMIBs, including the crystal structure, electrochemical performance, optimization strategies, and electrode reaction mechanisms. Meanwhile, recent research progress of AMIB full cells based on Mn-based oxide cathode materials is summarized. Finally, the challenges and future perspectives of Mn-based oxide cathode materials for AMIBs are discussed. This review will provide a valuable reference and source of inspiration for future research of manganese-based oxide cathode materials for AMIBs.

## 1. Introduction

The sustainable energy transition is an important path to the development of modern society, and advanced energy storage technology is the key to efficiently utilizing green, renewable energy [1]. Aqueous magnesium-ion batteries (AMIBs) are promising in next-generation energy storage due to multiple advantages: (1) The non-flammable nature of aqueous electrolytes makes AMIBs inherently safe [2,3,4,5]; (2) The high ionic conductivity of aqueous electrolytes bodes well for the rates [6,7]; (3) The abundant reserves of magnesium, low cost of aqueous electrolytes, and simple assembly conditions give AMIBs a significant cost advantage [8,9,10,11]; (4) AMIBs can potentially achieve a high energy density, if a magnesium metal anode with high theoretical capacity (3833 mAh cm^−3^ and 2205 mAh g^−1^) and low electrode potential (−2.37 vs. SHE) can be used [12,13,14,15]; (5) Both magnesium and aqueous electrolytes are environmentally benign. However, the development of AMIBs faces multiple challenges, and cathode materials constitute a key limiting factor. The electrochemical properties of cathode materials for AMIBs are typically consist of the slow diffusion kinetics of divalent Mg ions, the dissolution of transition metals in aqueous solutions, and the interfacial side reactions [16,17,18]. Therefore, significant research effort is being devoted to developing high-performance cathode materials for AMIBs.

Research on cathode materials for AMIBs has hitherto mainly focused on manganese-based oxides [17,18,19,20], vanadium-based oxides [4,19,20], and Prussian blue analogs [21,22,23]. For example, manganese-based oxides have attracted significant attention due to the abundant resources, low cost, and high theoretical capacity. Boasting rich oxidation states of manganese, such as +2, +3, and +4, there are many types of manganese-based oxides, such as MnO_2_ (oxidation state of +4), MgMn_2_O_4_ (oxidation state of +3), Mn_2_O_3_ (oxidation state of +3), Mn_3_O_4_ (oxidation state of +3 and +2), and MnO (oxidation state of +2) [24]. Meanwhile, MnO_2_ possesses various polymorphs [6]. The wide variety of species and crystal structures facilitates the regulation of electrochemical properties. However, pristine manganese-based oxides typically exhibit poor cycling stability and rate capability as a result of manganese dissolution, poor electronic conductivity, and sluggish Mg-ion diffusion kinetics [18,25,26]. Therefore, many optimization strategies, such as interlayer regulation [27,28], crystal defect engineering [26,29], heteroatom doping [30,31], and nanocomposite construction [25,32,33], have been proposed to improve the electrochemical properties, and significant progress has been made.

To develop high-performance manganese-based oxide cathode materials in the future, it is imperative to understand their crystal structure, electrode reactions and mechanisms, electrochemical properties, and optimization strategies. In this review, we aim to summarize the progress of Mn-based oxides as cathode materials for AMIBs. The crystal structures and classifications of Mn-based oxide cathode materials are discussed. Recent research on various Mn-based oxides is summarized, including their electrochemical properties, optimization, reaction mechanisms, and application to full cells. Finally, future perspectives of Mn-based oxide cathode materials for AMIBs are discussed.

## 2. Classification and Crystal Structure of Mn-Based Oxide Cathode Materials

**Figure 1:**
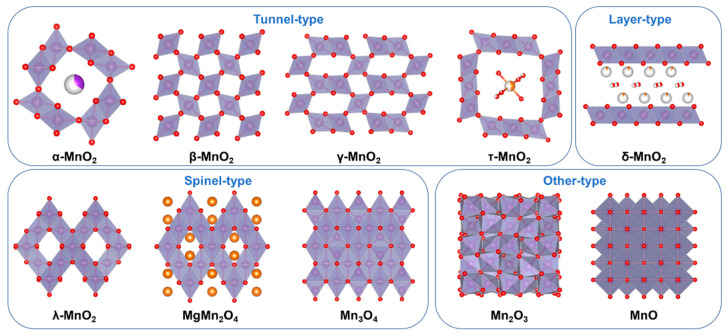
Classification and crystal structure of Mn-based oxide cathode materials.

Mn-based oxide cathode materials for AMIBs mainly include MnO_2_, MgMn_2_O_4_, Mn_3_O_4_, Mn_2_O_3,_ and MnO (Figure 1). Crystalline MnO_2_ is formed by connecting [MnO_6_] units based on edge-sharing or corner-sharing. Furthermore, different connecting modes produce various polymorphs, such as α, β, γ, δ, τ, and λ phases [6,24]. α-MnO_2_ has a [2 × 2] tunnel structure with a size of 4.6 × 4.6 Å, which facilitates the intercalation/deintercalation of Mg ions [31]. For α-MnO_2_, K ions are typically required as pillars in the channels to stabilize the tunnel structure. Although β-MnO_2_ is the most stable phase thermodynamically, it exhibits the narrowest [1 × 1] (2.3 × 2.3 Å) tunnel structure formed by corner sharing of [MnO_6_] units, being unfavorable to the intercalation/deintercalation of Mg ions [34]. γ-MnO_2_, the most common polymorph of manganese dioxide in the battery industry, has a mixed tunnel structure with [1 × 1] (2.3 × 2.3 Å) and [1 × 2] (2.3 × 4.6 Å) unit cells [35]. The crystal structure of ε-MnO_2_ is very similar to that of γ-MnO_2_, both of which are mixed tunnel structures, but ε-MnO_2_ has more structural defects [36,37]. Todorokite-MnO_2_ (τ-MnO_2_) has a [3 × 3] tunnel structure with the largest channel size (7 × 7 Å) [38]. However, as with α-MnO_2_, τ-MnO_2_ tunnels often contain a large number of impurity cations and water molecules to maintain structural stability. Unlike the aforementioned polymorphs with a tunnel structure, δ-MnO_2_ has a layered structure with an interlayer spacing of about 7 Å [27]. The large interlayer spacing and interlayer water of δ-MnO_2_ facilitate the diffusion and storage of Mg ions, thus δ-MnO_2_ has attracted a lot of attention in the field of AMIBs. λ-MnO_2_ is usually obtained from the spinel LiMn_2_O_4_ after removing lithium ions and inheriting the cubic spinel structure [39]. MgMn_2_O_4_ and Mn_3_O_4_ are manganese-based spinel oxides, often having distorted structures, on account of the Jahn–Teller effect of Mn^3+^ ions [40,41]. Mn_2_O_3_ primarily displays a crystal structure of an orthorhombic bixbyite at room temperature [42], and MnO exhibits a typical face-centered cubic structure [43]. In both structures, the Mg migration paths are blocked, rendering the materials unfavorable for Mg-ion insertion/extraction. In summary, based on the crystal structure, Mn-based oxide cathode materials can be classified into the following types (Figure 1): (1) Tunnel-type, such as α-MnO_2_ ([2 × 2] tunnel), β-MnO_2_ ([1 × 1] tunnel), γ-MnO_2_ ([1 × 2] tunnel), and τ-MnO_2_ ([3 × 3] tunnel); (2) Layer-type, such as δ-MnO_2_; (3) Spinel-type, such as λ-MnO_2_, MgMn_2_O_4_, and Mn_3_O_4_; (4) Other-type, such as Mn_2_O_3_ and MnO.

## 3. Recent Development of Mn-Based Oxide Cathode Materials for AMIBs

### 3.1. Tunnel-Type Mn-Based Oxides

#### 3.1.1. α-MnO_2_

As early as 2009, Xu et al. have investigated the electrochemical characteristics of α-MnO_2_ in an electrolyte containing Mg-ion (0.1 M Mg(NO_3_)_2_), observing ideal capacitive behavior with good cycling stability (5000 cycles) [44]. Sun et al. have reported that the capacitance of α-MnO_2_ in the 0.5 M MgSO_4_ electrolyte is lower than that in the 0.5 M Mg(NO_3_)_2_ electrolyte, but higher than that in the 0.25 M Na_2_SO_4_ or Al_2_(SO_4_)_3_ electrolyte [45]. Meanwhile, α-MnO_2_ with lower crystallinity exhibits better electrochemical properties. Zhang et al. have studied Mg-OMS-2 (Mg-α-MnO_2_) in different aqueous electrolytes (MgCl_2_, Mg(NO_3_)_2_, and MgSO_4_) and found better electrochemical properties in the Mg(NO_3_)_2_ electrolyte [46]. These results indicate that the electrolyte composition affects the electrochemical properties of α-MnO_2_.

To improve rate capability and cycling stability, some α-MnO_2_-based nanocomposites are used as cathode materials in AMIBs. For example, Zhang et al. have fabricated the Mg-OMS-2/graphene composite with better rate capability and cycling than Mg-OMS-2 [46]. In a 0.5 M Mg(NO_3_)_2_ electrolyte, the Mg-OMS-2/graphene composite exhibits a high capacity of 232.4 mAh g^−1^ at 20 mA g^−1^ and a capacity retention of 93.0% after 300 cycles at 100 mA g^−1^. Moreover, the transformation from the tetragonal phase to the cubic and hexagonal phases after Mg-ion intercalation is observed. Jia et al. have prepared vertically aligned α-MnO_2_ nanosheets on carbon nanotubes (α-MnO_2_/CNT) as cathode materials for AMIBs [47]. In the 1 M MgSO_4_ electrolyte, α-MnO_2_/CNT exhibits better capacity (144.6 mAh g^−1^) than α-MnO_2_ (87.5 mAh g^−1^) at 500 mA g^−1^, and even at 10,000 mA g^−1^, α-MnO_2_/CNT shows a capacity of about 60 mAh g^−1^ and a capacity retention of 85% after 1000 cycles.

**Figure 2:**
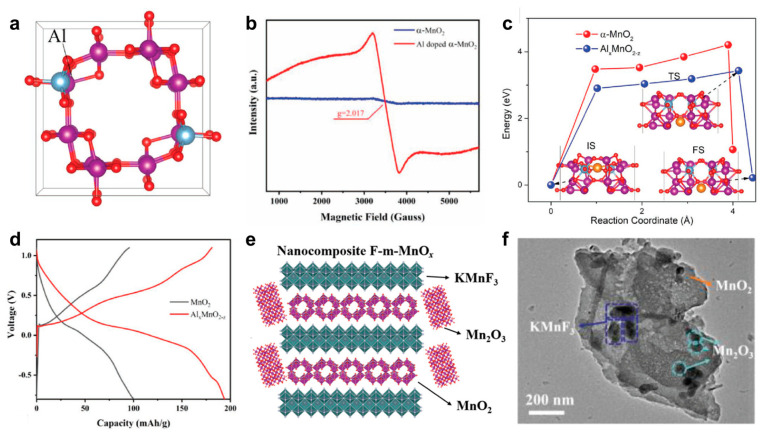
(**a**) Crystal structure of Al-doped α-MnO_2_, (**b**) electron paramagnetic resonance spectra, (**c**) calculated Mg-ion diffusion barriers, and (**d**) charging/discharging curves at 100 mA g^−1^ of α-MnO_2_ and Al-doped α-MnO_2_. Reproduced from [30], with permission under CC BY 4.0, (**e**) structural diagram and (**f**) TEM image of fluorine-regulated MnO_x_/KMnF_3_ heterostructure, reproduced from [25], with permission under CC BY 4.0.

Doping has been utilized to ameliorate the electrochemical properties of α-MnO_2_. Ding et al. have reported that partial substitution of Al for Mn in α-MnO_2_ (Figure 2a) creates more oxygen vacancies (Figure 2b) and regulates the diffusion of Mg ions (Figure 2c) [30]. Al shrinks the lattice due to the shorter length of the Al-O bond compared to the Mn-O bond, thereby improving the stability of α-MnO_2_ during ion insertion/extraction. As a result, Al-doped α-MnO_2_ exhibits a high capacity of 197.02 mAh g^−1^ at 100 mA g^−1^ (Figure 2d), good rate capability with a capacity of 90.5 mAh g^−1^ at 1000 mA g^−1^, and stable cycling with 82% retention after 2500 cycles. Wang et al. have found that Fe doping (15 mol%) enlarges the lattice of α-MnO_2_ and alleviates Jahn–Teller distortions, consequently improving Mg-ion diffusion kinetics and structural stability [31]. After Fe doping (15 mol%), the capacity of α-MnO_2_ at 2000 mA g^−1^ improves from 47.2 mAh g^−1^ to 99.6 mAh g^−1^, and the capacity retention after 1000 cycles increases from 44.3% to 85.2%. The reaction of α-MnO_2_ stems from the co-intercalation of Mg^2+^/H^+^. In addition, V-doped and Nb-doped α-MnO_2_ deliver improved rate performance in AMIBs, although their cycling stability needs further optimization [48]. Besides doping of Mn sites in α-MnO_2_, doping in tunnel sites (K sites) has been investigated. Zhao et al. have studied Mg-intercalation α-MnO_2_ with Mg ions in tunnel sites as cathode materials for AMIBs [49]. Compared to pristine α-MnO_2_, Mg-intercalation α-MnO_2_ exhibits an enhanced capacity of 419.8 mAh g^−1^ at 100 mA g^−1^ and retention of 111.6 mAh g^−1^ even at 4000 mA g^−1^. The in situ Raman spectra indicate the reversible phase transformation between α-MnO_2_ and δ-MnO_2_ during the charging/discharging process. However, a more detailed study of the electrode reaction mechanism of α-MnO_2_ is still needed. Recently, Gu et al. used F doping to enhance the magnesium storage capacity of α-MnO_2_ and prepared fluorine-regulated MnO_x_/KMnF_3_ heterostructures (Figure 2e,f) [25]. The high-strength Mn-F bonds formed in the MnO_2_ lattice locally enhance the structural stability by strengthening the tunnel structure and suppressing structural collapse during cycling. As a result, the fluorine-regulated MnO_x_/KMnF_3_ heterostructure achieves good cycling stability with a capacity retention of 89.6% after 1800 cycles at 1000 mA g^−1^.

#### 3.1.2. β-MnO_2_

Zhang et al. have evaluated the electrochemical performance of β-MnO_2_ (Mg-OMS-7) and investigated the size effect [34]. Compared to micro-sized β-MnO_2_, nano-sized β-MnO_2_ (1.6-Mg-OMS-7) has better electrochemical properties. Furthermore, 1.6-Mg-OMS-7 displays a high capacity of over 280 mAh g^−1^ at 10 mA g^−1^ in a 0.2 M Mg(NO_3_)_2_ electrolyte and a capacity retention of 94.1% after 200 cycles at 100 mA g^−1^ in the 1 M Mg(NO_3_)_2_ electrolyte. The electrochemical properties of 1.6-Mg-OMS-7 in Mg(NO_3_)_2_ and MgCl_2_ electrolytes are better than those in the MgSO_4_ electrolyte. Li et al. have improved the electrochemical properties of β-MnO_2_ in the MgSO_4_ electrolyte by regulating the nanostructure and Mn valence [50]. The nanostructure with a larger specific surface area exhibits a higher capacitive capacity, and a suitable Mn^3+^ concentration promotes the Faradic reaction. Therefore, β-MnO_2_ with a larger specific surface area and Mn^3+^ concentration of 16% exhibits a high capacity of 260 mAh g^−1^ at 200 mA g^−1^ in the 1 M MgSO_4_ electrolytes, and a capacity of 68 mAh g^−1^ at 10,000 mA g^−1^. However, cycling stability worsens since the capacity retention after 1000 cycles at 2000 mA g^−1^ is only 65%.

#### 3.1.3. τ-MnO_2_

Zhang et al. have studied τ-MnO_2_ (Mg-OMS-1) nanowires and nanosheets as cathode materials for AMIBs in different electrolytes [38,51]. In the 0.2 M MgCl_2_ electrolyte, τ-MnO_2_ nanosheets show a reversible capacity of 300 mAh g^−1^ at 10 mA g^−1^. However, the capacity of τ-MnO_2_ nanosheets decreases to 115 mAh g^−1^ when the current density increases to 100 mA g^−1^. To improve the properties of τ-MnO_2_, τ-MnO_2_/graphene composites [49] and Ni-doped τ-MnO_2_ [50] have been fabricated, but the improvements are limited.

#### 3.1.4. ε-MnO_2_

Liu et al. have synthesized binder-free ε-MnO_2_ nanoflakes on carbon cloth by potentiostatic electrodeposition [52]. Compared to commercial electrolytic MnO_2_, binder-free ε-MnO_2_ shows better capacity, rate performance, and cycling stability. In the 1 M MgCl_2_ electrolyte, a capacity of 259.3 mA h g^−1^ is achieved at 500 mA g^−1^. Moreover, reversible transformation between ε-MnO_2_ and MgMn_2_O_4_ occurs during discharging/charging. Furthermore, nanostructure regulation [53] and Fe doping [37] have been demonstrated to improve the electrochemical properties of ε-MnO_2_. However, compared to α-MnO_2_, research on other tunnel-type MnO_2_ for AMIBs is relatively limited.

### 3.2. Layer-Type Mn-Based Oxides

**Figure 3:**
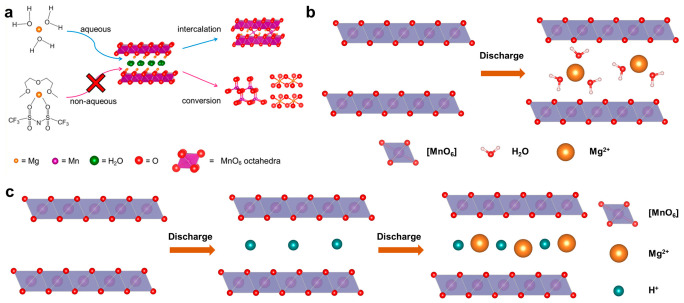
(**a**) Schematic of the magnesium storage reaction mechanism of δ-MnO_2_ in aqueous and non-aqueous electrolytes. Reproduced from [55] with permission. (**b**) Schematics of Mg^2+^/H_2_O co-insertion mechanism of δ-MnO_2_ in aqueous electrolyte. Adapted from [27], with substantial modifications. (**c**) Schematic of Mg^2+^/H^+^ co-insertion mechanism of δ-MnO_2_ in aqueous electrolyte. Adapted from [60], with substantial modifications.

Nam et al. have prepared Mg-δ-MnO_2_, also called Mg-birnessite MnO_2_ (Mg-B), from Mn_3_O_4_ using aqueous electrochemical transformation and evaluated its magnesium storage capacity [54]. In a 0.5 M Mg(ClO_4_)_2_ aqueous electrolyte, Mg-B displays a high capacity of 231.1 mAh g^−1^ at 100 mA g^−1^ and high rate with a capacity of 88.6 mAh g^−1^ at 2000 mA g^−1^. After 10,000 cycles at 2000 mA g^−1^, the capacity retention of Mg-B is 62.5%. Water in the electrolyte plays an important role in improving magnesium storage in Mg-B. In the Mg(ClO_4_)_2_/acetonitrile (ACN) electrolyte without water, Mg-B exhibits a low capacity of 56.8 mAh g^−1^ at 100 mA g^−1^. Sun et al. have demonstrated that magnesium storage in Mg-δ-MnO_2_ is based on the intercalation reaction in aqueous electrolyte and conversion reaction in a non-aqueous electrolyte (Figure 3a) [55]. In a 0.5 M Mg(ClO_4_)_2_ aqueous electrolyte, the interlayer spacing of Mg-δ-MnO_2_/CC contracts from 7 Å to 4.86 Å after Mg ion intercalation and expands to 10 Å after Mg ion extraction. Meanwhile, a glide of the MnO_2_ planes occurs during the intercalation/extraction of Mg ions. However, structural assessment of the discharged product (Mg-δ-MnO_2_ after Mg-ion intercalation) is inadequate. The interlayer spacing change in δ-MnO_2_ during intercalation/de-intercalation of Mg ions reported in many subsequent studies is smaller than that reported by Sun et al. [29,56,57,58,59]. The intercalation species in δ-MnO_2_ as cathode materials in AMIBs is also an unanswered question worth exploring. Based on the electrochemical quartz crystal microbalance (EQCM), Wang et al. have proposed that some water molecules are intercalated into δ-MnO_2_ along with magnesium ions (Figure 3b) [27]. Liu et al. have verified the consequent H^+^ and Mg^2+^ insertion reaction in δ-MnO_2_ by comparing the electrochemical properties of δ-MnO_2_ in different aqueous electrolytes and Mg concentrations at different charge/discharge states (Figure 3c) [60]. During the discharge process, the H^+^ insertion occurs first at a slightly higher potential, followed by magnesium ion insertion. The intercalation species tend to be hydrated H^+^ and hydrated Mg^2+^, but more evidence is needed. Moreover, the effect of electrolyte composition on the Mg^2+^/H^+^ co-insertion is also unclear.

**Figure 4:**
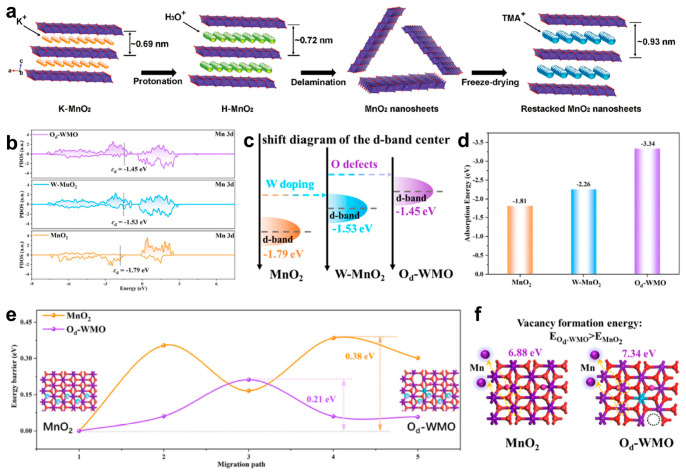
(**a**) Schematic of the synthetic process and crystal structure of TMA-MnO_2_. Reproduced from [63] with permission. (**b**) Mn 3*d*-band center values of δ-MnO_2_, W-doped δ-MnO_2_ (W-MnO_2_), and O_d_-WMO, (**c**) shift diagram of the Mn 3*d*-band center, (**d**) Mg-ion adsorption energy, (**e**) Mg-ion diffusion barriers and (**f**) energy barrier for Mn vacancy formation in δ-MnO_2_ and O_d_-WMO. Reproduced from [29], with permission under CC BY 4.0.

Interlayer regulation is an efficient strategy to improve the electrochemical performance of layered materials, and this is no exception for δ-MnO_2_ in AMIBs. δ-MnO_2_ with different interlayer cations (Li^+^, Na^+^, K^+^, and Mg^2+^) has been investigated as cathode materials for AMIBs [28,54,61,62]. Huang et al. have compared the electrochemical properties of δ-MnO_2_ with different interlayer cations (Li^+^, Na^+^, and K^+^) synthesized by the same electrodeposition method [28]. K^+^-intercalated δ-MnO_2_ exhibits a higher capacity and rate performance. Xiong et al. have fabricated tetramethyl ammonium (TMA^+^) ions-intercalated δ-MnO_2_ (TMA-MnO_2_) with an expanded interlayer spacing of 0.93 nm (Figure 4a) and investigated the metal-ion (Li^+^, Na^+^, K^+^, and Mg^2+^) storage behavior [63]. Compared to pristine δ-MnO_2_ (K-MnO_2_) with an interlayer spacing of 0.69 nm and protonated δ-MnO_2_ (H-MnO_2_) with an interlayer spacing of 0.72 nm, TMA-MnO_2_ has better electrochemical characteristics. Compared with Na^+^ and K^+^ storage, TMA-MnO_2_ shows a higher capacity for Mg^2+^ storage at a low current density, albeit with poorer rate performance. The magnesium storage capacity retention of TMA-MnO_2_ is as high as 80% after 5000 cycles at 5000 mA g^−1^. Moreover, the water content interlayer plays an important role in the magnesium storage capacity of δ-MnO_2_. Sun et al. have studied two δ-MnO_2_ materials with different interlayer water contents [56]. Compared to Mg_0.61_MnO_2_·0.42H_2_O (MMO-2), Mg_0.58_MnO_2_·0.56H_2_O (MMO-1), with a higher interlayer water content, has a larger interlayer space (∼9.70 Å), which is favorable to the diffusion of Mg ions. Consequently, MMO-1 shows a better rate performance. In a 0.5 M MgCl_2_ electrolyte, MMO-1 exhibits a capacity of 98.3 mAh g^−1^ at 1000 mA g^−1^, which is higher than that of MMO-2 (74.4 mAh g^−1^).

Nanostructure regulation is an effective approach to improve the properties of materials [64], which has also been used for δ-MnO_2_ in AMIBs. Shi et al. have fabricated hierarchical Mg-birnessite nanowall arrays with enriched (010) planes (ECMB) in situ by electrochemical conversion from Mn_3_O_4_ nanowall arrays [58]. Compared to Mg-birnessite nanosheets prepared by a traditional hydrothermal synthesis, ECMB has a smaller nanosheet size and enriched active (010) planes, facilitating fast Mg^2+^ intercalation/deintercalation. ECMB shows significant improvements in capacity and rate performance. In the 0.5 M Mg(ClO_4_)_2_ aqueous electrolyte, ECMB exhibits a high capacity of 255.1 mAh g^−1^ at 200 mA g^−1^, which remains at 55.4 mAh g^−1^ at 8000 mA g^−1^. Moreover, EMCB exhibits significantly improved cycling stability, with a capacity retention of 73.6% after 3000 cycles at 2000 mA g^−1^. Some δ-MnO_2_-based nanocomposites have been synthesized as cathode materials for AMIBs, for example, δ-MnO_2_@carbon molecular sieves (CMS) composite [65], δ-MnO_2_/multiwalled carbon nanotubes (MWCNTs) composite [57,66], δ-MnO_2_/reduced graphene oxide (rGO) composite [67], and K-δ-MnO_2_/mulberry-like carbon (K-MnO_2_/HMC) composite [68]. Compared to the corresponding pristine δ-MnO_2_, these composites have better electrochemical properties. For example, δ-MnO_2_@CMS shows a high capacity of ~270 mAh g^−1^ at 50 mA g^−1^ in a 0.5 M Mg(NO_3_)_2_ electrolyte, which remains at 140 mAh g^−1^ at 1000 mA g^−1^ [65]. However, the cycling stability needs improvement.

Recently, crystal defect engineering has been adopted to optimize the electrochemical properties of δ-MnO_2_ in AMIBs. Ren et al. have synthesized oxygen-deficient W-doped δ-MnO_2_ (O_d_-WMO) as cathode materials [29]. The combination of W doping and oxygen defects leads to the upward shift in the *d*-band center of Mn (Figure 4b,c), the narrowing of the bandgap, and the strengthening of Mn-O bonds (Figure 4f), thereby improving the adsorption capacity for Mg^2+^ (Figure 4d), charge transfer, ion transport kinetics (Figure 4e), and structural durability (Figure 4f). O_d_-WMO shows a high capacity (185.2 mAh g^−1^ at 100 mA g^−1^), enhanced rate performance (101.2 mAh g^−1^ at 2000 mA g^−1^), and good cycling stability (capacity retention of 92% after 1500 cycles). Suitable Fe doping improves the capacity and rate capability of δ-MnO_2_ [31]. δ-MnO_2_ with 5 mol% Fe exhibits a high capacity of 263.5 mAh g^−1^ at 100 mA g^−1^, which retains 145.8 mAh g^−1^ at 2000 mA g^−1^. However, a larger Fe concentration leads to a phase transformation from the δ- to α-phase.

Anionic doping is another effective technique. Zhang et al. have synthesized F-doped δ-MnO_2_ (MnO_2−x_F_y_) from K_1.33_Mn_8_O_16_ by thermal treatment with NH_4_F using it as a cathode material for AMIBs [69]. During the thermal process, many oxygen vacancies are introduced into MnO_2−x_F_y_, and the morphology changes from nanorods to a three-dimensional hierarchical structure. Compared to K_1.33_Mn_8_O_16_, MnO_2−x_F_y_ exhibits improved specific capacitance and rate performance. The combination of crystal defect engineering and nanocomposite construction further improves the electrochemical properties of δ-MnO_2_. Liu et al. have fabricated V-doped and O vacancy-rich δ-MnO_2_/CNTs/rGO (V-O_vac_-MnO_2_/CG) composites [59]. V doping promotes the generation of O vacancies to lower the electrostatic barrier for ion transport and enhance the electronic conductivity. Meanwhile, CNTs and rGO improve the electrical conductivity. Therefore, the V-O_vac_-MnO_2_/CG composite achieves a high discharge capacity of 398 mAh g^−1^ at 100 mA g^−1^ and good cycling stability with a capacity retention of 81% after 500 cycles at 200 mA g^−1^. Even at 2000 mA g^−1^, the V-O_vac_-MnO_2_/CG composite exhibits a reversible capacity of 143 mAh g^−1^. Layered A_x_MnO_2_ (A = Na or K) without interlayer water has been investigated, but the electrochemical properties are poorer than those of δ-MnO_2_ [70,71], demonstrating that the water interlayer plays an important role in the superior electrochemical performance of δ-MnO_2_.

### 3.3. Spinel-Type Mn-Based Oxides

#### 3.3.1. λ-MnO_2_

Yuan et al. have synthesized λ-MnO_2_ nanoparticles from spinel LiMn_2_O_4_ by acid leaching to remove Li and determined the electrochemical properties in aqueous electrolytes containing different Mg salts (MgSO_4_, MgCl_2_, and Mg(NO_3_)_2_) [39]. λ-MnO_2_ has better properties in the 1 M MgCl_2_ electrolyte, as manifested by a high discharge capacity of ~480 mAh g^−1^ at 13.6 mA g^−1^. When cycling at 136 mA g^−1^, the capacity of λ-MnO_2_ remains at 155.6 mAh g^−1^ after 300 cycles. Tekin et al. have reported that λ-MnO_2_ delivers better cycling performance in the 1 M MgSO_4_ electrolyte than 1 M MgCl_2_ and 1 M Mg(NO_3_)_2_ electrolytes [72]. λ-MnO_2_ is detached from the current collectors in MgCl_2_ and Mg(NO_3_)_2_ electrolytes but not in MgSO_4_. In addition, λ-MnO_2_ can be prepared from MgMn_2_O_4_ using an acid treatment, but MgMn_2_O_4_ can be directly used in the cathode of AMIBs [40]. Therefore, most λ-MnO_2_ used for AMIBs is still prepared from LiMn_2_O_4_. Chen et al. have investigated the effect of particle size on magnesium storage in λ-MnO_2_ [73]. Compared to micron-sized λ-MnO_2_, nano-sized λ-MnO_2_ has a higher reversible capacity, especially the capacity contributed from Faradaic process. The phase transition from the cubic to the tetragonal spinel structure during Mg-ion intercalation is studied by X-ray diffraction (XRD) and atomic-resolution scanning transmission electron microscopy (STEM) imaging. Zhang et al. have synthesized λ-MnO_2_/MWCNTs [74]. In a 0.5 M MgSO_4_ electrolyte, the λ-MnO_2_/MWCNTs composite displays an initial discharge capacity of 124.1 mAh g^−1^ at 1000 mA g^−1^ and capacity retention of 86.2% after 1000 cycles.

#### 3.3.2. MgMn_2_O_4_

Sinha et al. have synthesized MgMn_2_O_4_ from LiMn_2_O_4_ by electrochemical conversion and determined the electrochemical properties in the 1 M Mg(NO_3_)_2_ aqueous electrolyte [75]. However, the obtained MgMn_2_O_4_ exhibits a low discharge capacity of only 42 mAh g^−1^. MgMn_2_O_4_ can be synthesized by the sol–gel method, hydrothermal method, and other techniques. MgMn_2_O_4_ nanoparticles directly synthesized by Cabello et al. by the sol–gel method show a discharge capacity of 150 mAh g^−1^ in a 3 M Mg(NO_3_)_2_ electrolyte [40]. Unfortunately, the capacity decays below 100 mAh g^−1^ after 20 cycles, indicating poor cycling stability.

To improve the electrochemical properties of MgMn_2_O_4_ in AMIBs, several MgMn_2_O_4_-based nanocomposites have been prepared. For example, Liu et al. have fabricated MgMn_2_O_4_ nanoparticles/rGO (MgMn_2_O_4_/rGO) composite [33]. Compared to MgMn_2_O_4_ nanoparticles, MgMn_2_O_4_/rGO exhibits a higher capacity and rate. In a 0.5 M MgCl_2_ electrolyte, the specific capacity of MgMn_2_O_4_/rGO is 211.8 mAh g^−1^ at 50 mA g^−1^ and remains at 140.1 mAh g^−1^ at 1000 mA g^−1^. Moreover, a capacity of 119.5 mAh g^−1^ is observed after 1000 cycles at 1000 mA g^−1^. Javadian et al. have synthesized MgMn_2_O_4_ hollow spheres/rGO composite and evaluated its electrochemical properties in a 1 M MgSO_4_ mixed electrolyte of water/acetonitrile (1:10 in volume) [76]. The composite exhibits a high capacity of 305 mAh g^−1^ at 300 mA g^−1^ with a capacity retention of ~95% after 100 cycles. Zhang et al. have synthesized the MgMn_2_O_4_/MWCNTs composites from LiMn_2_O_4_/MWCNTs by electrochemical conversion [77,78]. The MgMn_2_O_4_/MWCNTs composites obtained from commercial LiMn_2_O_4_ exhibit a high capacity of over 400 mAh g^−1^ at 50 mA g^−1^, good cycling stability with a retention rate of 73.3% after 1000 cycles at 1000 mA g^−1^ [77]. However, the Coulombic efficiency of MgMn_2_O_4_/MWCNTs composites is low, especially at low current density, indicating that irreversible reaction or side reaction may occur during the discharge process. Recently, MgMn_2_O_4_/polyaniline (PANI) has been prepared as a cathode material for AMIBs [79]. The optimized MgMn_2_O_4_/PANI composite delivers a high capacity of 287 mAh g^−1^ at 250 mA g^−1^ and 73% capacity retention after 250 cycles at 500 mA g^−1^.

**Figure 5:**
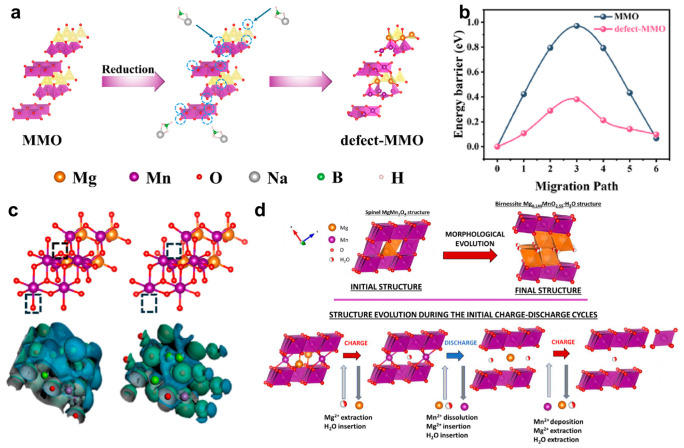
(**a**) Synthesis of oxygen-deficient MgMn_2_O_4_ (defect-MMO), (**b**) Mg-ion diffusion energy barriers, and (**c**) structure and charge density difference distribution diagrams of MgMn_2_O_4_ (MMO) and defect-MMO. Reproduced from [26], with permission under CC BY 4.0. (**d**) Schematic of the structural evolution from spinel MgMn_2_O_4_ to a layered structure during the electrochemical process. Reproduced from [83], with permission under CC BY 4.0.

Cationic substitution is an effective route to enhance the structural stability and electrochemical kinetics of LiMn_2_O_4_ and it is also excepted to improve the electrochemical properties of MgMn_2_O_4_ in AMIBs. In this case, Zhang et al. have synthesized MgFe_x_Mn_2−x_O_4_ (x = 0.67, 1, 1.33, 1.6) nanoparticles as cathode materials for AMIBs [80]. MgFe_1.33_Mn_0.67_O_4_ (MFM-2) has better electrochemical characteristics, including higher capacity, enhanced rate capability, and cycling stability. MFM-2 exhibits a high capacity of 88.3 mAh g^−1^ after 1000 cycles at 1000 mA g^−1^ without obvious attenuation. Compared to MgMn_2_O_4_, MgMn_1.5_Ni_0.5_O_4_ has better cycling stability [81]. After 500 cycles at 1000 mA g^−1^, the capacity retention of MgMn_1.5_Ni_0.5_O_4_ is 92.9%, but that of MgMn_2_O_4_ is only about 30%. The properties of MgMn_1.5_Ni_0.5_O_4_ can be further improved using a polypyrrole (PPy) coating [82], which improves not only the electrical conductivity but also the structural stability by alleviating expansion/contraction and Mn dissolution. Consequently, the optimized MgMn_1.5_Ni_0.5_O_4_@PPy composite exhibits a high capacity of 133.0 mAh g^−1^ with 90.8% capacity retention after 2000 cycles at 1000 mA g^−1^. Zhao et al. have incorporated oxygen vacancies into MgMn_2_O_4_ (Figure 5a) [26]. DFT calculations indicate that oxygen vacancies reduce the Mg^2+^ diffusion barrier (Figure 5b), narrow the bandgap, and generate favorable adsorption sites for Mg^2+^ by inducing charge redistribution (Figure 5c). Therefore, the optimized oxygen-deficient MgMn_2_O_4_ exhibits an improved capacity of 230.8 mAh g^−1^ at 100 mA g^−1^ and retains a capacity of 84 mAh g^−1^ at 2000 mA g^−1^.

The electrochemical reaction mechanism of MgMn_2_O_4_ in AMIBs remains controversial, with three mechanisms proposed. (1) Tetragonal spinel MgMn_2_O_4_ transforms to cubic spinel λ-MnO_2_ after Mg-ion extraction during charging, and the transformation is reversible [40,77,78]. (2) Tetragonal spinel MgMn_2_O_4_ transforms irreversibly to the birnessite-type layered phase during early cycling (Figure 5d) [83,84]. (3) Tetragonal spinel MgMn_2_O_4_ irreversibly transforms into MnO_2_ (α- or γ-phase) after Mg-ion extraction during charging, and MnO_2_ undergoes a combined reaction involving intercalation from MnO_2_ to MgMnO_2_ and conversion from MnO_2_ to MgO and MnO during discharging [76,79]. The evidence supporting the third mechanism is limited, consisting only of poor-quality ex situ XRD data (Table 1), therefore it may be a flawed conclusion. The complete spinel to layer transformation (second mechanism) typically requires dozens of cycles [84], but most mechanistic studies only focus on the first or first two cycles, thus failing to observe the spinel to layer transformation. Before transforming into a layered structure, MgMn_2_O_4_ may undergo reversible magnesium ion extraction (first mechanism). These may be the reasons why different studies report different results. To determine the more precise electrode reaction mechanism of MgMn_2_O_4_ further research is needed.

Compared to the tetragonal spinel MgMn_2_O_4_, the cubic inverse-spinel Mg_2_MnO_4_ containing a higher concentration of Mn^4+^ and is expected to expedite ion diffusion. Sun et al. have studied Mg_2_MnO_4_ [85]. In the 1 M MgSO_4_ + 0.1 M MnSO_4_ electrolyte, the Mg_2_MnO_4_ exhibits a capacity of 116.1 mAh g^−1^ at 1C (160 mA g^−1^), which remains 60.8 mAh g^−1^ even at 20 °C (3200 mA g^−1^), demonstrating good rate performance. However, the theoretical capacity of Mg_2_MnO_4_ is only 160 mAh g^−1^ (based on the Mn^4+^/Mn^3+^ redox couple), which is much lower than that of other manganese oxides and is unfavorable for achieving higher energy density.

**Table 1:** The proposed reaction mechanisms for different Mn-based oxides.

Mn-Based Oxides	Proposed Reaction Mechanisms	Evidences	Ref.
α-MnO_2_	Mg^2+^ insertion	Ex situ XRD, XPS, HRTEM	[25,47]
α-MnO_2_	Mg^2+^/H^+^ co-insertion	Ex situ XRD, XPS, HRTEM, DFT calculations	[31]
α-MnO_2_	Mg^2+^ insertion with partial transformation to new cubic phase and hexagonal phase	Ex situ XRD	[46]
α-MnO_2_	Mg^2+^ insertion with reversible transformation to layered birnessite	In situ Raman	[49]
β-MnO_2_	Mg^2+^ insertion	Ex situ XRD, XPS	[34,50]
τ-MnO_2_	Mg^2+^ insertion with partial transformation to layered birnessite	Ex situ XPS, XRD	[51]
τ-MnO_2_	Mg^2+^ insertion	Ex situ XPS, XRD	[38,86]
ε-MnO_2_	Mg^2+^ insertion with reversible transformation to MgMn_2_O_4_	Ex situ XPS, XRD and EDS	[52]
δ-MnO_2_	Mg^2+^ insertion	In situ Raman, Ex situ XRD, XPS, HRTEM, ICP, Raman, STEM, EDS, EELS	[29,56,57,58,59,63,65,66,87,88]
δ-MnO_2_	Mg^2+^/H_2_O co-insertion	EQCM	[27]
δ-MnO_2_	Mg^2+^/H^+^ co-insertion	GITT, ex situ XPS, EDS	[60]
δ-MnO_2_	Mg^2+^ insertion with reversible transformation to unknown new phase	Ex situ XRD	[55]
λ-MnO_2_	Mg^2+^ insertion with cubic to tetragonal phase transformation	Ex situ XRD, STEM, XPS	[40,73,74]
MgMn_2_O_4_	Mg^2+^ extraction	Ex situ XRD, XPS	[26,80]
MgMn_2_O_4_	Mg^2+^ extraction with tetragonal to cubic phase transformation	Ex situ XRD	[77,78]
MgMn_2_O_4_	Irreversible transformation to layered birnessite during the initial cycling	In situ Raman, ex situ XRD, HRTEM, TG-DTA, FT-IR, SEM, XPS	[80,84]
MgMn_2_O_4_	Irreversible transformation to MnO_2_ (α- or γ-phase) during Mg-ion extraction and a combined reaction involving insertion to MgMnO_2_ and conversion to MgO and MnO during discharging.	Ex situ XRD	[76,79]
Mn_3_O_4_	Irreversible transformation to layered birnessite during the initial cycling	Ex situ XRD, HRTEM	[41,86]
Mn_3_O_4_	Mg^2+^ insertion	Ex situ XRD, XPS	[88]
Mn_3_O_4_	Mg^2+^ insertion with Mn(II) dissolution induced tetrahedral vacancies as insertion sites	Ex situ XRD, HRTEM, XANES, XPS, Raman	[18]
Mn_2_O_3_	Mg^2+^ insertion	Ex situ XRD, XPS	[32,89]
MnO	Mg^2+^ insertion	Ex situ XRD, XPS	[90]

#### 3.3.3. Mn_3_O_4_

Besides Mg-Mn spinel oxides, spinel Mn_3_O_4_ has been investigated as the cathode material in AMIBs. In an aqueous electrolyte containing Mg^2+^, Mn_3_O_4_ transforms to birnessite-MnO_2_ (δ-MnO_2_), accompanied by Mn^2+^ dissolution during the electrochemical process [89]. Therefore, some δ-MnO_2_ materials are obtained from Mn_3_O_4_ by the electrochemical conversion method discussed in Section 3.2 [27,54,58]. Pan et al. have discovered that Mn_3_O_4_ nanoparticles electrochemically transform into the amorphous-crystalline Mn_3_O_4_ composite (Mn_3_O_4_-A) when cycling in the NaSO_4_ aqueous electrolyte [91]. Moreover, as a cathode material in AMIBs with the MgSO_4_ aqueous electrolyte, Mn_3_O_4_-A (crystalline part) retains its tetragonal spinel structure during cycling rather than transforming into a layered structure. In addition, although Mn_3_O_4_ transforms to δ-MnO_2_ during cycling, its initial structural characteristics still have a significant impact on the electrochemical performance. Ding et al. have fabricated Mn_3_O_4_ with cation-anion dual defects (CADDs-Mn_3_O_4_) and better properties than commercial Mn_3_O_4_ [41]. CADDs-Mn_3_O_4_ shows a high capacity of 320.93 mAh g^−1^ at 100 mA g^−1^ in the 0.5 M MgSO_4_ electrolyte. Moreover, CADDs-Mn_3_O_4_ exhibits a capacity of ~200 mAh g^−1^ even after 2000 cycles at 1000 mA g^−1^. Li et al. have found that introducing a small amount of Cs in the hydrothermal process can induce the phase transition from Mn_2_O_3_ to Mn_3_O_4_, thereby obtaining Cs-doped Mn_3_O_4_ [90]. In a 0.5 M MgSO_4_ aqueous electrolyte, Cs-doped Mn_3_O_4_ has better properties than Mn_2_O_3_. However, it is uncertain whether the electrochemical improvement stems from Cs doping or a phase transition.

**Figure 6:**
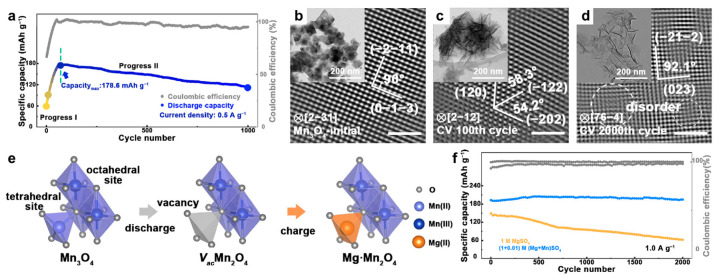
(**a**) Cycling performance of Mn_3_O_4_, (**b**–**d**) TEM images and low-pass filtered HR-TEM images of Mn_3_O_4_ after different CV cycles, (**e**) schematic diagram of the crystal structure evolution of Mn_3_O_4_, (**f**) cycling performance of Mn_3_O_4_ in electrolytes with and without the MnSO_4_ additive. Reproduced from [18], with permission under CC BY 4.0.

Pan et al. have investigated Mn dissolution and structure transformation in Mn_3_O_4_ during cycling [18]. Dissolution of Mn(II) during the electrochemical process causes the morphological transformation (Figure 6b–d) and the formation of tetrahedral vacancies (Figure 6e). The vacancies not only expedite Mg-ion diffusion but also provide storage sites. During initial cycling, the capacity of Mn_3_O_4_ increases with cycles (Figure 6a). Notably, the spinel framework remains the same, rather than transforming into a layered structure during 4000 CV cycles at 25 mV s^−1^. Furthermore, the collapse of the spinel framework may be responsible for the fading capacity during subsequent cycling. Adding MnSO_4_ to the electrolyte can stabilize the spinel framework by inhibiting the dissolution of Mn(III) via the common-ion effect (Figure 6f). Therefore, the capacity retention of Mn_3_O_4_ after 2000 cycles at 1000 mA g^−1^ increases from 42% to 94.9% after adding 0.1 M MnSO_4_ into the electrolyte.

### 3.4. Other Mn-Based Oxides

Other Mn-based oxides, such as Mn_2_O_3_ and MnO, have been employed as cathode materials for AMIBs. In order to promote Mg-ion transport kinetics, Li et al. have constructed Mn_2_O_3_@TiO_2_@MXene composites with dual heterogeneous interfaces, thereby creating built-in electric fields and reducing the diffusion energy barrier [92]. The Mn_2_O_3_@TiO_2_@MXene composites achieve an improved capacity of 241.5 mAh g^−1^ in a 0.5 M MgSO_4_ aqueous electrolyte compared to Mn_2_O_3_. After 1350 cycles at 1000 mA g^−1^, the Mn_2_O_3_@TiO_2_@MXene composites still exhibit a capacity retention of 85.7%. An organic-inorganic coupling strategy has been proposed to improve magnesium-ion storage in Mn_2_O_3_ by fabricating organic-inorganic ethylenediamine-Mn_2_O_3_ (EDA-Mn_2_O_3_) composites [32]. EDA adsorbed on the surface of Mn_2_O_3_ can capture Mg ions by the coordination effect of the terminal biprotonic amine, promoting the electrochemical reaction kinetics. Consequently, EDA-Mn_2_O_3_ delivers enhanced capacity, rate performance, and cycling stability. Meanwhile, the reversible structural evolution of Mn_2_O_3_ during Mg-ion insertion/extraction is confirmed. Liu et al. reported the MnS/MnO heterostructure with dual-ion defects (DID-MnS/MnO) for aqueous magnesium-ion storage [93]. Compared to MnS, DID-MnS/MnO exhibits a highly enhanced capacity of 237.9 mAh g^−1^ at 100 mA g^−1^ and maintains a capacity retention of 94.7% after 1200 cycles at 1000 mA g^−1^.

### 3.5. Electrolyte Effects

The electrolyte, as the ion transport medium between cathode and anode, has a decisive influence on the ion migration kinetics, electrochemical stability window, electrode/electrolyte interface characteristics, and reaction mechanism of the battery system. Therefore, the design and optimization of the electrolyte system is crucial for the practical application of manganese-based oxide cathode materials for AMIBs.

The electrolyte composition has a significant impact on the solvation structure of magnesium ions, thereby affecting the desolvation energy barrier. For example, the SO_4_^2−^ anion exhibits a significantly positive Jones-Dole B coefficient, classifying it as a strongly hydrated anion, which makes it difficult to competitively replace water molecules in the first solvation shell of magnesium ions, resulting in a stable, dense Mg[(H_2_O)_6_]^2+^ configuration [46,94]. The abundant coordinated water and strong Mg–O bonds significantly raise the desolvation energy barrier. In contrast, Cl^−^ and NO_3_^−^ anions have lower Jones-Dole B coefficients (mostly negative), indicating weaker self-hydration, thereby they can partially replace water molecules in the first solvation shell of magnesium ions, effectively reducing the desolvation energy barrier. Therefore, Mn-based oxide cathode materials usually displays lower capacity and poor rate performance in aqueous MgSO_4_ electrolytes than in aqueous MgCl_2_ and Mg(NO_3_)_2_ electrolytes with same concentration.

In addition, the electrolyte composition design can regulate the proton activity of the system, which significantly affect the proton insertion or co-insertion behavior [95,96]. Usually, the capacity contribution from proton insertion decreases with the increase in pH (decrease in proton activity). Meanwhile, the proton activity will affect the dissolution behavior of the cathode or anode materials [97,98]. However, the specific correlation between the proton activity of the electrolyte and the electrochemical performance of the manganese-based oxide cathode materials for AMIBs still lacks systematic research.

Mn dissolution is a key bottleneck limiting the electrochemical performance of Mn-based oxide cathode materials for AMIBs. Optimizing electrolyte composition is an effective strategy to inhibit this problem. Adding an appropriate amount of MnSO_4_ into an electrolyte can effectively suppress manganese dissolution by the common ion effect [18,85]. In addition, by adding appropriate electrolyte additives, a protective electrode-electrolyte interphase can be formed in situ, which can also effectively inhibit the manganese dissolution [99].

Although conventional dilute aqueous electrolytes exhibit high ionic conductivities, their electrochemical stable window (ESW) is restricted by the theoretical decomposition voltage of H_2_O (1.23 V). Broadening the ESW of aqueous electrolytes is key to improving the operating voltage and energy density of AMIBs. Recently, some electrolytes with expanded ESW have been designed for AMIBs, including super-concentration (water-in-salt) electrolyte [100], eutectic electrolyte [101], organic solvent-in-water electrolyte [102], and quasi-solid-state electrolyte [103]. However, the electrochemical properties of Mn-based oxide cathode materials in these electrolytes still need further systematic study.

## 4. Full Cells Based on Mn-Based Oxide Cathode Materials

The research on full-cell batteries is an important step towards the practical application of manganese-based oxide cathode materials for AMIBs. Currently, anode materials used to assemble full cells with Mn-based oxide cathode materials are mainly organic compounds, such as 3,4,9,10-perylenetetracarboxylic dianhydride (PTCDA), 3,4,9,10-perylenetetracarboxylic diimide (PTCDI), and polyimide (PI), as well as vanadium-based oxides like VO_2_, FeVO_4_, and VO*_x_*. Some studies employ activated carbon anodes paired with manganese oxide cathodes, but the resulting devices are magnesium-ion hybrid capacitors rather than AMIBs. Therefore, these devices will not be discussed in this section. To achieve higher energy density, some researchers have paired Mg metal anodes with Mn-based oxide cathode materials for full-cell assembly, but this places greater demands on the electrolyte. In this section, the recent progress of AMIB full cells is discussed.

Vanadium-based oxide anode materials have been widely used in AMIBs due to their suitable electrode potential and high theoretical capacity. Zhang et al. have assembled the FeVO_4_·0.9H_2_O/graphene||Mg-OMS-1 (τ-MnO_2_) full battery with the 1 M MgSO_4_ electrolyte, exhibiting a discharge capacity of 65.1 mAh g^−1^ and energy density of 58.5 Wh kg^−1^ at 50 mA g^−1^ [104]. In this section, capacity and energy density are based on the total active mass of the cathode and anode, unless otherwise specified. The anode materials are changed from FeVO_4_·0.9H_2_O/graphene to FeVO_4_/C during the assembly of the FeVO_4_/C||Mg-OMS-1 full battery, which shows a discharge capacity of 78.2 mAh g^−1^ and energy density of 70.4 Wh kg^−1^ [105]. The results demonstrate that anode materials also play an important role in AMIB full cells. Compared to FeVO_4_, VO_2_ is used more extensively in AMIBs because of its higher capacity, which is favorable for achieving higher energy density. For example, the VO_2_||δ-MnO_2_@CMS full battery exhibits a higher capacity of 112.2 mAh g^−1^ [65]. Subsequently, various AMIB full cells based on the VO_2_ anode and Mn-based oxide cathode have been assembled, for instance, VO_2_||δ-MnO_2_/MWCNT [57], VO_2_||Ni-doped τ-MnO_2_ [106], VO_2_||Li_0.21_MnO_2_·H_2_O (Li-δ-MnO_2_) [61], VO_2_||MgMn_2_O_4_/MWCNTs [78], VO_2_||MgMn_1.5_Ni_0.5_O_4_ [81], and so on. In order to improve the properties of the VO_2_||δ-MnO_2_ full battery, Li et al. have proposed an over-discharge pretreatment strategy [87]. After the over-discharge treatment, both Mg-δ-MnO_2_ and Ca/Mg-δ-MnO_2_ cathode materials are controllably cleaved into ultrafine nanoparticles. The difference is that Mg-δ-MnO_2_ becomes amorphous, while the layered structure of Ca/Mg-δ-MnO_2_ is retained. This is attributed to the pillar effect of pre-intercalated Ca-ion in Ca/Mg-δ-MnO_2_. The VO_2_||Ca/Mg-δ-MnO_2_ full battery after the pretreatment delivers an enhanced capacity of 113.2 mAh g^−1^ and an improved rate (45.8 mAh g^−1^ even at 1000 mA g^−1^). Moreover, a high capacity retention of 72.5% is achieved after 1000 cycles at 50 mA g^−1^. Meanwhile, the over-discharge treatment of VO_2_||δ-MnO_2_ full battery using an electrolyte containing Cl^−^ (2 M Mg(NO_3_)_2_ + 25 mM MgCl_2_) produces different results [107]. After the over-discharge pretreatment with Cl^−^ in the electrolyte, the δ-MnO_2_ turns into amorphous ultrafine nanoparticles, but a crystalline V_6_O_13_ layer is generated on the surface. Meanwhile, the VO_2_ anode is transformed into polycrystalline vanadium oxide containing mixed-valence states (+4 and +5). The generation of the V_6_O_13_ layer is attributed to the dissolution of vanadium species from the vanadium-based anode into the electrolyte mediated by chloride ions. Therefore, the full-cell capacity is enhanced to 120.62 mAh g^−1^. However, the Coulombic efficiency (below 90%) and cycling stability (37.52% retention after 700 cycles) are unsatisfactory. The low-temperature electrochemical properties of the AMIB full battery composed of Mn-based oxide cathode materials are evaluated. Yang et al. have reported that the VO_2_||δ-MnO_2_ full battery with the 4 M MgCl_2_ aqueous electrolyte can work in a wide temperature range from +25 to −50 °C [108]. At −20 °C, the full battery capacity is 97.9 mAh g^−1^ at 100 mA g^−1^ and 25.5 mAh g^−1^ at 3000 mA g^−1^, and after 1000 cycles at 2000 mA g^−1^, 90% capacity retention is achieved. A flexible quasi-solid-state AMIB based on the polyacrylamide/MgCl_2_ hydrogel electrolyte exhibits exciting properties, including low-temperature performance, mechanical flexibility, and safety.

Organic compounds constitute another important class of anode materials for AMIBs. Liu et al. have assembled the PTCDA||ε-MnO_2_ full battery with 1 M MgCl_2_ electrolyte, which exhibits good rates (246.6 mAh g^−1^ at 500 mA g^−1^ and 63.3 mAh g^−1^ at 6000 mA g^−1^, based on the mass of the cathode material) and cycling stability (capacity retention of 72.6% after 800 cycles) [52]. The energy density of the PTCDA||ε-MnO_2_ full battery is 98.6 Wh kg^−1^. Compared to PTCDA, PTCDI typically exhibits better cycling stability in aqueous batteries, which is beneficial for the full battery. Therefore, more researchers have paired the PTCDI anode with the Mn-based oxide cathode in the AMIB full battery. Various AMIB full cells based on the PTCDI anode and Mn-based oxide cathode have been reported, such as PTCDI||MgMn_2_O_4_ [84], PTCDI||MgMn_2_O_4_/rGO [67], PTCDI||Mg-intercalation α-MnO_2_ [49], PTCDI||MnO_2−x_F_y_ [69], PTCDI||O_d_-WMO [29], and PTCDI||Mn_3_O_4_[18]. Among these, the PTCDI||MgMn_2_O_4_ full battery using a 0.5 M Mg(NO_3_)_2_ electrolyte delivers an energy density of 80.9 Wh kg^−1^ at 648 W kg^−1^ (based on the mass of cathode material) and a capacity retention of 80.9% even after 5000 cycles at 1000 mA g^−1^ [84]. The PTCDI||MnO_2−x_F_y_ full battery with the 1 M Mg (NO_3_)_2_ electrolyte achieves a high energy density of 106.1 Wh kg^−1^ at 0.5 kW kg^−1^ and a capacity retention of 88.1% even after 10,000 cycles at 5000 mA g^−1^ [69]. Adding manganese salts to the electrolyte can inhibit manganese dissolution, thereby improving the cycle performance of manganese-based oxide cathode materials and AMIB full cells [18,85]. The PI||Mg_2_MnO_4_ full battery using 1 M MgSO_4_ + 0.1 MnSO_4_ electrolyte has good rate performances and a high capacity retention of 89% after 10,000 cycles at 100 C (18,000 mA g^−1^) [85]. In contrast, the PI||Mg_2_MnO_4_ full battery with 1 M MgSO_4_ electrolyte, without MnSO_4_, exhibits poor rate capability and rapid capacity decay.

**Figure 7:**
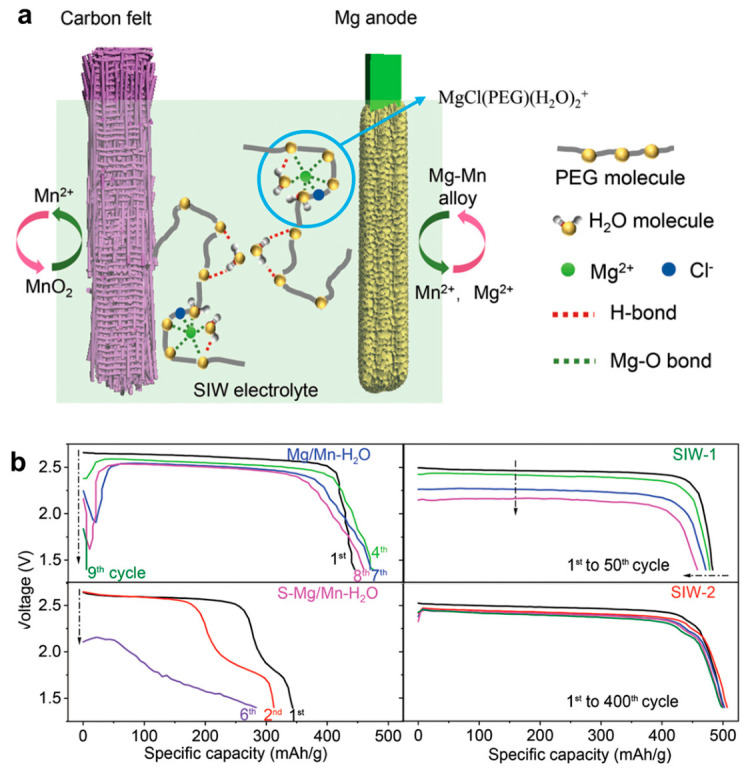
(**a**) Schematic illustration of the structure and working mechanism of Mg||MnO_2_ battery using the SIW electrolyte and (**b**) Discharge curves of Mg||MnO_2_ batteries with different electrolytes. Reproduced from [102], with permission.

Although some AMIBs with full cells based on Mn-based oxide cathodes and V-based or organic anodes exhibit good rate performance and cycling stability, their energy density is limited by the low average discharge voltage (typically not exceeding 1 V). Mg metal is the ultimate negative electrode sought after for aqueous magnesium-ion batteries because it can achieve high operating voltage and high energy density, but it undergoes serious side reactions and passivation in traditional aqueous electrolytes. Xu et al. have proposed an organic solvent-in-water electrolyte (saturated MgCl_2_ + 1 m MnCl_2_ in H_2_O:poly(ethylene glycol) 400 = 1:1, denoted as SIW-2) to avoid the passivation of Mg metal anode, and a high-voltage and stable aqueous Mg||MnO_2_ battery (Figure 7a) is obtained [102]. The Mg||MnO_2_ battery using SIW-2 electrolyte displays a discharge plateau at ~2.5 V and a discharge capacity of ~500 mAh g^−1^ (based on the mass of MnO_2_ on the cathode) (Figure 7b) with a capacity retention of around 99% after over 1000 cycles. The work demonstrates the feasibility of constructing high-performance AMIB full cells using Mg metal anodes and Mn-based oxide cathodes.

Energy density is one of the most important indicators for batteries in practical application. Most reported energy density of AMIB full cells are calculated merely based on the active mass of cathode or the total active mass of cathode and anode, whereas practical energy density should take the total weight of the cathode, anode, electrolyte, separator, current collectors and packaging into consideration. However, most reported AMIB full cells have low active materials loading, excessive electrolyte dosage and limited capacity (mAh-level), resulting in extremely low practical energy density. Therefore, improving the practical energy density requires not only enhancing the specific capacity and potential gap between cathode and anode materials, but also elevating active material loading, optimizing electrolyte dosage and developing high-capacity (Ah-level) pouch-cell configurations. For future practical application, the initial goal of AMIBs should be to compete with lead-acid batteries. When the practical energy density of AMIBs exceeds 40 Wh/kg, it will demonstrate significant comprehensive competitiveness due to its advantages such as environmental friendliness and abundant reserves. Currently, there is still a long way to go.

## 5. Conclusions and Outlook

Manganese-based oxides are an important class of cathode materials for AMIBs due to the abundant resources, low cost, high theoretical capacity, and environmental friendliness. Manganese-based oxide cathode materials for AMIBs are reviewed, including the tunnel type (α-MnO_2_, β-MnO_2_, γ-MnO_2_, and τ-MnO_2_), layer type (δ-MnO_2_), spinel-type (λ-MnO_2_, MgMn_2_O_4_, and Mn_3_O_4_), and other types (Mn_2_O_3_ and MnO) of manganese-based oxides. The crystal structure, electrochemical properties, optimization strategies, and electrode reaction mechanisms of various manganese-based oxide cathode materials are discussed. By using various efficient optimization strategies, including interlayer regulation, crystal defect engineering, heteroatom doping, and nanocomposite construction, many optimized manganese-based oxides achieve significant improvements in electrochemical performance (Table 2). Meanwhile, many AMIB full cells based on manganese-based oxide cathode materials have been reported (Table 3), and some of them show good rate capability and cycling stability. However, despite recent advances, the further development of Mn-based oxide cathode materials for AMIBs remains challenging.

Although the electrochemical properties of Mn-based oxide cathode materials for AMIBs have been improved by various optimization techniques, they still cannot meet the demands of practical applications. Mn dissolution, poor electronic conductivity, and slow Mg-ion diffusion kinetics are still the major issues. The Mn dissolution causes fast capacity fading, while poor electronic conductivity and slow Mg-ion diffusion kinetics limit the performance rate. For further improvements, research on other aqueous batteries, especially aqueous zinc-ion batteries, can provide valuable insights. In addition, artificial intelligence (AI)-assisted design based on machine learning and prediction may be helpful in developing high-performance Mn-based oxide cathode materials for AMIBs. However, the challenge lies in obtaining reliable data sources and building suitable learning models. In addition, the practical application potential of these optimization strategies needs further consideration. The nanostructure or nanocomposite construction cannot solve the slow magnesium-ion diffusion kinetics and will lead to a series of issues including reduced tap density, increased specific surface area causing more side reactions, and easy agglomeration. Interlayer regulation, crystal defect engineering, and heteroatom doping are all optimization strategies at the crystal level that can effectively improve the magnesium-ion diffusion kinetics in the lattices of Mn-based oxides. However, interlayer regulation is only applicable to layered materials, and increasing the interlayer spacing leads to a decrease in tap density. In contrast, doping and crystal defect engineering seem more suitable for future practical applications, and some doping will also induce crystal defects.

Understanding the electrode reaction mechanism of Mn-based oxide cathode materials is crucial to optimizing their electrochemical properties. In this respect, the electrode reaction mechanism of some manganese-based oxide cathode materials is still unclear or controversial (Table 1). For example, whether the intercalated species are Mg ions, protons, or both. Meanwhile, the different electrolytes and operating conditions may lead to different electrode reaction mechanisms. Therefore, more in-depth investigations are required.

To develop high-performance AMIBs based on Mn oxide cathodes, research on anode materials and electrolytes is also crucial. Anode materials with lower electrode potentials and high capacity are key to achieving high energy density. Mg metal is the ultimate negative electrode sought after, but it undergoes serious side reactions and passivation in traditional aqueous electrolytes. Therefore, electrolyte limitation is the biggest challenge for developing AMIB full cells using Mn-based oxide cathodes and Mg metal anodes. Excitingly, recent work demonstrates that electrolyte engineering and the design of artificial solid electrolyte interphases are effective approaches to alleviate these issues. However, more research is needed to achieve better improvement results.

Moreover, there is a gap between laboratory research and practical application. The mass loading of Mn-based oxide cathode materials in most previous literature is less than 5 mg cm^−2^ (Table 2), which is too low for practical applications. Even if the specific capacity of manganese-based oxide cathode materials reaches 300 mAh g^−1^, the areal capacity of commercial lithium-ion batteries (over 3.0 mAh cm^−2^) would still require a mass loading exceeding 10 mg cm^−2^. However, the electrochemical properties of manganese-based oxides for AMIBs at high mass loadings (>10 mg cm^−2^) remain poorly understood. It is worth noting that some progress has been made in high mass loading manganese oxide cathodes for zinc-ion batteries, which can provide a reference for manganese-based oxide cathode materials for AMIBs [109]. Meanwhile, a pouch-cell demonstration of AMIBs full cell with manganese-based oxide cathode materials has been reported, but the capacity of the reported pouch cells is relatively low. The demonstration of high-capacity pouch cells (e.g., Ah-level) is very important for assessing the practical application potential of manganese-based oxide cathode materials. To achieve higher energy density, parameters such as active material loading, electrolyte-to-capacity ratio, and N/P ratio need to be considered. The long-term cycling stability under practical conditions is also important. In addition, prior to commercial application, exploring the large-scale synthesis of the high-performance manganese-based oxide cathode materials is necessary.

In summary, although manganese-based oxide cathode materials have great potential for AMIBs, much more work is needed to deepen our understanding of them, achieve further breakthroughs, and ultimately bring them to commercial fruition. Meanwhile, this review serves as a reference and a source of inspiration for future research on manganese-based oxide cathode materials for AMIBs.

**Table 2:** Electrochemical properties of various Mn-based oxide cathode materials for AMIBs.

Materials	Electrolyte	Active-Material Mass Loading (mg cm^−2^)	Reference Electrode	Potential Range (V, vs. RE)	Capacity (mAh g^−1^)/Current Density (mA g^−1^)	Cycle Number/Remained Capacity (mAh g^−1^)/Retention Rate/Current Density (mA g^−1^)	Ref.
α-MnO_2_/graphene	0.5 M Mg(NO_3_)_2_	~5.0	SCE	−0.65–0.75	232.4/20, 115/100	300/-/93.0%/100	[46]
α-MnO_2_/CNT	1.0 M MgSO_4_	-	SCE	−0.6–1.4	144.6/500, 59.7/10,000	1000/48.3/85%/10,000	[47]
Mg-intercalation α-MnO_2_	1.0 M MgCl_2_	-	Graphite	−0.6–1.0	~419.8/100, 111.6/4000	1000/-/95.4%/1000	[49]
Al-doped α-MnO_2_	0.5 M MgSO_4_	-	Hg/HgO	−0.8–1.1	197.02/100, 90.50/1000	2500/-/82%/1000	[30]
10% Nb-doped α-MnO_2_	1.0 M Mg(NO_3_)_2_	~3.5	SCE	−0.6–0.9	252.6/20, 168.7/100	200/65.1/38.6%/100	[48]
10% V-doped α-MnO_2_	1.0 M Mg(NO_3_)_2_	~3.5	SCE	−0.6–0.9	265.9/20, 178.7/100	200/79.4/36.8%/100	[48]
15 mol% Fe-doped α-MnO_2_	1 M MgCl_2_	~2.0	Ag/AgCl	−0.65–1.0	205.9/100, 171.8/200, 118.6/500, 101/1000, 99.6/2000	1000/84.9/85.2%/2000	[31]
Fluorine-regulated MnO_x_/KMnF_3_	0.5 M MgSO_4_	~1.3	Hg/HgO	−0.8–1.1	142/100, 106/200, 85.2/300, 73.5/500, 60.9/800, 54.8/1000	1800/-/89.6%/1000	[25]
β-MnO_2_ (Mg-OMS-7) nanorods	0.2 M Mg(NO_3_)_2_	~7.0	SCE	−0.65–0.75	283.1/10, 102/100	200/97.21/95.3%/100	[34]
β-MnO_2_ porous nanoflowers	1 M MgSO_4_	~2.0	Ag/AgCl	−0.2–1.0	260/200, 151/1000, 68/10,000	1000/169/65%/2000	[50]
τ-MnO_2_ (Mg-OMS-1) nanobelts	0.5 M Mg(NO_3_)_2_	~7.0	SCE	−0.65–0.8	243/10, 149/20, 93.5/100	200/-/90.4%/100	[38]
τ-MnO_2_ (Mg-OMS-1) nanosheets	0.2 M MgCl_2_	~7.0	SCE	−0.65–0.8	300/10, 115/100	300/-/86%/100	[51]
τ-MnO_2_ (Mg-OMS-1)/graphene	0.5 M Mg(NO_3_)_2_	-	SCE	−0.65–0.8	194.1/20, 100.1/100	200/92.5/92.5%/100	[86]
Ni-doped τ-MnO_2_	0.5 M Mg(NO_3_)_2_	6.0–7.0	SCE	−0.65–0.75	318/10	300/-/89.7%/100	[106]
ε-MnO_2_ on carbon cloth	1.0 M MgCl_2_	2.0	Ag/AgCl	−0.65–1.0	259.3/500, 114.4/2000	400/-/94.3%/2000	[52]
ε-MnO_2_ nanoflowers	1.0 M MgSO_4_	1.0	SCE	−0.5–1.3	230.9/1000	-	[53]
Li-δ-MnO_2_ nanowires	0.5 M Mg(NO_3_)_2_	-	SCE	−0.65–0.75	226.7/20, 165.8/100	300/93.4/56.3%/100	[61]
K-δ-MnO_2_ nanoparticles	1 M MgSO_4_	~1.5	Ag/AgCl	−0.2–1.1	163/100, 185/1000, 78/10,000	1000/160/86.7%/1000	[62]
δ-MnO_2_ nanoflowers	1.0 M MgCl_2_	2.0	Ag/AgCl	−0.65–1.0	252.1/50, 198.2/100, 150.8/200, 125.4/500, 109.7/1000, 95.2/2000	800/-/54.4%/1000	[60]
δ-MnO_2_ from γ-MnS	1.0 M Mg(NO_3_)_2_	~1.5	Ag/AgCl	−0.2–1.0	~360/616	50/~200/-/308	[87]
Mg-δ-MnO_2_	0.5 M Mg(ClO_4_)_2_	-	Ag/AgCl	−0.3–1.1	231.1/100, 216.8/200, 172.4/500, 138.4/1000, 112.3/2000	500/-/78.3%/500, 10,000/-/62.5%/2000	[54]
Mg-δ-MnO_2_ with larger interlayer space (∼9.70 Å)	0.5 M MgCl_2_	~6.0	SCE	−0.8–1.0	169.3/50, 153.2/100, 98.3/1000	100/~150/-/100	[56]
Mg-δ-MnO_2_/carbon cloth	0.5 M Mg(ClO_4_)_2_	~1.5	Ag/AgCl	−0.3–1.3	~150/500	160/~45/-/500	[55]
TMA-MnO_2_	1.0 M MgSO_4_	-	Ag/AgCl	−0.6–0.9	110.8/232.69, 50.3/2326.9	500/63.3/-/465.38	[27]
δ-MnO_2_ nanosheet arrays on Ti foi	0.5 M Mg(ClO_4_)_2_	-	Ag/AgCl	−0.3–1.1	250/100, 210/200, 150/500, 135/1000, 90/2000	1500/63/84%/3000	[110]
ECMB	0.5 M Mg(ClO_4_)_2_	1.0–2.0	Ag/AgCl	−0.2–1.3	255.1/200, 203.5/500, 166.4/1000, 122.7/2000, 85.6/4000, 55.4/8000	3000/-/73.6%/2000	[58]
δ-MnO_2_@CMS	0.5 M Mg(NO_3_)_2_	~3.0–5.0	SCE	−0.6–0.8	270.3/50	300/~136/60.5%/50	[65]
δ-MnO_2_/MWCNT	0.5 M MgSO_4_	~3.9	SCE	−0.7–1.0	313.2/50, 189.9/100, 56.8/1000	500/-/~100%/1000	[57]
δ-MnO_2_@MWCNTs/CC	0.5 M MgSO_4_	4.4	SCE	−0.7–1.0	246.7/50, 75.2/1000	2000/60.5/80%/1000	[66]
δ-MnO_2_/rGO composite	2 M MgSO_4_ + 2 M MgOAc	-	Ag/AgCl	0–1.0	88.5/1000, 80.2/2000, 74.9/3000, 70.8/4000, 67.3/5000	-	[67]
K-MnO_2_/HMC	1.0 M MgSO_4_	~1.0	Hg/HgO	−0.5–1.3	168/500, 132/1000, 89/3000, 69/5000, 52/8000, 45.3/10,000	-	[68]
K^+^-intercalated δ-MnO_2_ on graphitic nanofibers	1.0 M MgSO_4_	1.5	Ag/AgCl	0–0.8	91.18/50, 51.88/4000	-	[28]
MnO_2−x_F_y_ (F-doped δ-MnO_2_)	1.0 M Mg(NO_3_)_2_	~2.0	SCE	0–1.0	491.5 F g^−1^/500, 123.5 F g^−1^/20,000	10,000/-/83.9%/5000	[69]
O_d_-WMO	1.0 M MgSO_4_	-	Ag/AgCl	−0.3–1.1	185.2/100, 153.8/200, 133.5/500, 116.9/1000, 101.2/2000	1500/-/92%/500	[29]
V-O_vac_-MnO_2_/CG	0.5 M MgSO_4_	~3.1	SCE	−0.82–0.85	398/100, 295/200	500/239/81%/200	[59]
Micron-sized Na_0.7_MnO_2.05_	1 M MgSO_4_ + 0.008 M sodium lauryl sulfate	6.0–8.0	SCE	0–0.8	40/20, 35/40, 22/100, 13/200	1000/10.2/59.8%/200	[70]
λ-MnO_2_ nanoparticles	1.0 M MgCl_2_	~7.0	SCE	−0.8–1.0	478.4/13.6, 288.0/136, 253.9/408	300/155.6/-/136	[39]
λ-MnO_2_ nanoparticles	1.0 M MgSO_4_	~2.5–3.0	SCE	−0.8–0.4	220/0.025C, 180/0.05C, 161/0.1C, 88/0.2C	100/-/52.5%/0.1C	[72]
λ-MnO_2_ nanoparticles	1.0 M Mg(NO_3_)_2_	~3.0	Ag/AgCl	−0.2–1.0	352/27, 293/90, 217/270, 110/1350	100/110/59%/270	[73]
λ-MnO_2_/MWCNTs	0.5 M MgSO_4_	3.5–5.0	SCE	−0.7–1.0	213.8/50, 127.2/1000	1000/-/86.2%/1000	[74]
MgMn_2_O_4_ form LiMn_2_O_4_ ^#^	1.0 M Mg(NO_3_)_2_	4.0–6.0	SCE	−0.6–0.5	42/45.5	20/35/-/45.5	[75]
MgMn_2_O_4_ nanoparticles	3.0 M Mg(NO_3_)_2_	2.1–3.7	Ag/AgCl	0–1.3	~150/60 μA	20/~90/-/60 μA	[40]
MgMn_2_O_4_/rGO nanocomposites	0.5 M MgCl_2_	~5.5	SCE	−0.5–0.85	211.8/50, 140.1/1000	1000/119.5/85.3%/1000	[33]
MgFe_1.33_Mn_0.67_O_4_	0.5 M MgCl_2_	~6.0	SCE	−0.8–1.1	136.5/50, ~88.3/1000	1000/~88.3/100%/1000	[80]
MgMn_2_O_4_/MWCNTs	0.5 M MgSO_4_	-	SCE	−0.7–1.0	412.9/50, 318.4/100, 276.3/200, 212/300, 186.8/500, 176.6/800, 163.2/1000	1000/106.3/73.3%/1000	[77]
MgMn_2_O_4_/MWCNTs	0.5 M MgSO_4_	~4.0	SCE	−0.7–1.0	322.3/50, 239.1/100, 207.8/200, 189.8/300, 169.6/500, 152.8/800, 145.8/1000	2000/101/81.8%/1000	[78]
MgMn_2_O_4_ hollow sphere/rGO	1 M MgSO_4_ in water/acetonitrile (1:10 in volume)	-	Ag/AgCl	−0.4–1.2	305/300	100/290/-/300	[76]
MgMn_2_O_4_/PANI	0.5 M MgSO_4_	~0.2–1.0	Ag/AgCl	−0.6–1.2	232/500	250/170/73%/500	[79]
MgMn_1.5_Ni_0.5_O_4_@PPy	0.5 M MgCl	-	SCE	−0.6–0.9	273.3/50, 150.2/1000	2000/133/90.8%/1000	[82]
MgMn_1.5_Ni_0.5_O_4_	0.5 M MgCl	-	SCE	−0.6–0.9	221.8/50, 186.5/100, 162.2/200, 142.5/300, 136.8/500, 121.8/800, 115.2/1000	500/104/92.9%/1000	[81]
MgMn_2_O_4_ with Oxygen defects	0.5 M MgSO_4_	1.68	SCE	−0.2–1.0	230.8/100, 84/2000	550/~110/-/500	[26]
Mg_2_MnO_4_	1.0 M MgSO_4_ + 0.1 M MnSO_4_	1.0–2.0	Ag/AgCl	−0.2–0.8	116.1/160, 71.7/800, 60.8/3200	50/71.7/-/800	[85]
Milling-Mn_3_O_4_	2.0 M MgSO_4_	~5.0	Ag/AgCl	−0.2–1.0	105.8/100, 93.2/500, 79.8/1000, 68.5/2000	300/81/-/500	[89]
Mn_3_O_4_-A	1.0 M MgSO_4_	-	SCE	0–1.3	98.9/200, 73.8/500, 61.5/1000, 50.0/2000, 33.1/5000	2000/~98.3/99.4%/200	[91]
CADDs-Mn_3_O_4_	0.5 M MgSO_4_	~1.4	Hg/HgO	−0.6–1.0	392.93/100, 181.24/1000	2000/181.24/100%/1000	[41]
Cs-doped Mn_3_O_4_	0.5 M MgSO_4_	~1.3	Hg/HgO	−0.8–1.1	214.44/100, 179.60/200, 154.90/300, 131.30/500, 116.20/800, 110.14/1000	1500/-/95.38%/1000	[90]
Mn_3_O_4_ nanoparticles	1 M MgSO_4_ + 0.01 M MnSO_4_	-	SCE	−0.5–1.4	311/200, 105.6/5000	2000/-/94.9%/1000	[18]
Mn_2_O_3_@TiO_2_@MXene	0.5 M MgSO_4_	~1.3	Hg/HgO	−0.8–1.1	241.5/100, 180.5/200, 154.6/300, 138.3/500, 114.4/800, 106.7/1000	1350/-/85.7%/1000	[92]
EDA-Mn_2_O_3_	0.5 M MgSO_4_	-	Hg/HgO	−0.8–1.1	188.97/100, 160.47/200, 143.02/300, 128.47/500, 110.79/800, 97.24/10,000	1200/-/93.63%/1000	[32]
DID-MnS/MnO	0.5 M MgSO_4_	~1.3	Hg/HgO	−0.8–1.1	237.9/100, 161.8/200, 141.3/300, 117.8/500, 101.7/800, 95.9/1000	1200/90.1/94.7%/1000	[93]

^#^ We believe that the unit of current density in the original paper is wrong, it should be mA/g instead of A/g.

**Table 3:** Electrochemical properties of AMIB full cells constructed with Mn-based oxide cathode materials.

Full Battery	Electrolyte	Voltage Range (V)	Average Discharge Voltage (V)	Capacity (mAh g^−1^)/Current Density (mA g^−1^)	Cycle Number/Capacity/Retention Rate/Current Density (mA g^−1^)	Energy Density (Wh kg^−1^)/Power Density (W kg^−1^)	Ref.
FeVO_4_/C||Mg-OMS-1 (τ-MnO_2_)	1.0 M MgSO_4_	0.0–1.8	~0.8	78.2/50, 58.9/100, 49.5/200, 38.9/300, 32.1/500	100/-/97.7%/100	70.4/-	[105]
FeVO_4_·0.9H_2_O/graphene||Mg-OMS-1 (τ-MnO_2_)	1.0 M MgSO_4_	0.0–1.8	~0.6	65.1/50, 53.1/100, 42.3/200, 32.0/300, 24.4/500	100/-/97.2%/100	58.5/50.8	[104]
VO_2_||δ-MnO_2_@CMS	1.0 M MgSO_4_	0.0–1.8	~0.9	112.2/50, 94.6/100, 78.7/200, 69.5/300, 59.1/500	100/-/46.9%/-	-	[65]
VO_2_||Ni-doped τ-MnO_2_ ^#^	1.0 M MgSO_4_	0.0–1.8	~0.9	198.2/50, 125.1/100, 102.7/200, 95/300, 81.9/500	100/-/94.6/100	-	[106]
VO_2_||δ-MnO_2_/MWCNT	0.5 M MgSO_4_	0.0–2.0	~0.7	108.4/50,81.5/100	500/20/-/1000	-	[57]
VO_2_||Li-δ-MnO_2_	0.5 M MgSO_4_	0.0–1.6	~0.7	74.8/20, 54.7/50, 46.4/100, 34.0/200, 32.1/300	50/46.2/80.5%/100	-	[61]
VO_2_||MgMn_2_O_4_/MWCNTs	0.5 M MgSO_4_	0.0–1.9	~0.8	115/50, 75.8/100, 54.0/200, 41.5/300, 30.7/500, 21.4/800, 17.6/1000	500/18.1/~100%/1000	-	[78]
VO_2_||δ-MnO_2_ ^#^	4 M MgCl_2_	0.0–1.8	~0.7	228.5/100 (25 °C), 97.9/100(−20 °C), 37.1/100 (−50 °C)	1000/-/74%/2000 (25 °C), 1000/-/90%/2000 (−20 °C), 1000/-/~100%/2000 (−50 °C)	-	[108]
VO_2_||δ-MnO_2_ ^#^	PAAm/MgCl_2_ hydrogel electrolyte	-	-	178.5/100 (25 °C), ~80/100(−20 °C)	500/-/88%/2000 (25 °C), 500/-/~100%/2000 (−20 °C)	-	[108]
VO_2_/V_6_O_13_||V_6_O_13_@Mg-MnO_2_ ^#^	2 M Mg(NO_3_)_2_ + 25 mM MgCl_2_	−0.45–1.4	~0.3	120.62/50	100/117.55/97.45%/50, 200/86.8/71.96%/50, 700/45.21/37.52%/50	-	[107]
VO_2_||MgMn_1.5_Ni_0.5_O_4_ *	0.5 M MgCl	0.0–1.7	~0.7	85.8/50, 24.8/1000	400/22.1/82.1%/1000	-	[81]
VO_2_||nano-Mg/Ca-δ-MnO_2_ *	2 M Mg(NO_3_)_2_ + 0.01 M MgSO_4_	−0.45–1.4	~0.3	113.2/50, 94.85/100, 74.6/250, 59.1/500, 45.8/1000	1000/63.39/72.5%/50, 500/-/87.7%/500	-	[88]
VO_x_||MnO_2_ *	4.5 M MgCl_2_	~0.0–1.8	~0.8	144.9/130, 73.9/260, 59.9/400, 54.2/670, 47/1300, 40.3/2600	1000/-/84.5%/2600	-	[111]
V_6_O_13−x_||δ-MnO_2_ *	1.0 M Mg(Ac)_2_	0.01–1.6	~0.7	55.1/200, 131.2/500, 122.6/700, 111.5/1000, 92.1/2000	200/123/~80%/200, 3000/45/50%/2000	-	[97]
PTCDA||ε-MnO_2_ ^#^	1.0 M MgCl_2_	~0.0–1.7	~0.7	246.6/500, 160/1000, 63.3/6000	800/-/72.6%/1000	98.6/-	[52]
PI||Mg_2_MnO_4_ ^#^	1 M MgSO_4_ + 0.1 M MnSO_4_	0.0–1.6	~0.8	93.6/360, 70.7/18,000	10,000/60.7/89%/18,000	60.1/15,300	[85]
PI||ECMB *	0.5 M Mg(ClO_4_)_2_	0.0–2.2	~0.9	65/100, 33/1000	500/-/99%/500	65.2/96	[58]
PTCDI||MgMn_2_O_4_ ^#^	0.5 M Mg(NO_3_)_2_	0.01–2.10	~0.7	194/100, 125/1000	5000/124.8/80.9%/1000	80.9/648	[84]
PTCDI||Mg-intercalated α-MnO_2_ *	1 M MgCl_2_	-	-	94.8/200, 82.6/500, 77.3/800, 74.8/1000, 69.5/2000, 64.4/4000	400/-/103.44%/-	-	[49]
PTCDI||δ-MnO_2_/rGO *	2 M MgSO_4_ +2 M MgOAc	0.0–2.0	~0.8	36.2/1000, 35.2/2000, 33.8/3000, 32.1/4000, 30.6/5000	3000/-/76.4%/2000	29.8/822.5, 25/4098.36	[67]
PTCDI||MnO_2−x_F_y_	1.0 M Mg(NO_3_)_2_	0.0–2.0	~1.0	190.9 F g^−1^/500, 66.1 F g^−1^/10,000	10,000/-/88.1%/5000	106.1/0.5, 15.9/20	[69]
PTCDI||Mn_3_O_4_ *	1 M MgSO_4_ + 0.01 MnSO_4_	0.0–2.7	~1.0	73.3/200, 63.2/500, 52.4/1000, 44.8/2000	1000/-/88.8%/1000	75.9/211.6, 42.8/1772.7	[18]
PTCDI||O_d_-WMO *	1.0 M MgSO_4_	0.0–1.5	~0.3	106.6/100, 96.5/200, 82.6/500, 70.6/1000, 60.2/2000	1500/-/83%/500	80.4/75	[29]
Mg||MnO_2_ ^#^	Saturated MgCl_2_ + 1 M MnCl_2_ in H_2_O:PEG = 1:1 (SIW-2)	1.4–2.8	~2.5	~500/5C (1 mA cm^−2^)	1000/-/~99%/5C (1 mA cm^−2^)	-	[102]

Calculations of capacity and energy density are based on the total active mass of the cathode and anode, unless otherwise specified. ^#^ Specific capacities or energy density/power densities are calculated based on the mass of cathode material. * The mass on which the calculation of specific capacity or energy density/power density is based is not mentioned.

## Data Availability

Not applicable.
